# Venturing in coral larval chimerism: a compact functional domain with fostered genotypic diversity

**DOI:** 10.1038/srep19493

**Published:** 2016-01-13

**Authors:** Baruch Rinkevich, Lee Shaish, Jacob Douek, Rachel Ben-Shlomo

**Affiliations:** 1 Israel Oceanographic and Limnological Research, National Institute of Oceanography, Tel Shikmona, P.O. Box 8030, Haifa 31080, Israel; 2 Department of Evolutionary and Environmental Biology, Faculty of Natural Sciences, University of Haifa, Mount Carmel, 31905, Israel; 3 Department of Biology, Faculty of Natural Sciences, University of Haifa–Oranim, Tivon 36006, Israel

## Abstract

The globally distributed coral species *Pocillopora damicornis* is known to release either sexual or asexual derived planula-larvae in various reef locations. Using microsatellite loci as markers, we documented the release of asexually derived chimeric larvae (CL), originating from mosaicked maternal colonies that were also chimeras, at Thai and Philippines reefs. The CL, each presenting different combinations of maternal genotypic constituents, create genetically-complex sets of asexual propagules. This novel mode of inheritance in corals challenges classical postulations of sexual/asexual reproduction traits, as asexual derived CL represent an alliance between genotypes that significantly sways the recruits’ absolute fitness. This type of inherited chimerism, while enhancing intra-entity genetic heterogeneity, is an evolutionary tactic used to increase genetic-heterogeneity, primarily in new areas colonized by a limited number of larvae. Chimerism may also facilitate combat global change impacts by exhibiting adjustable genomic combinations of within-chimera traits that could withstand alterable environmental pressures, helping *Pocillopora* become a successful cosmopolitan species.

Life-history strategies, among them sexual and asexual reproductive traits, reveal sets of co-adapted demographic and evolutionary tactics that determine the population dynamics and absolute fitness of specific organisms in any given environment. Variations in reproductive strategies among the populations of any species may influence genetic parameters and population genetic features^1^. The literature further reveals numerous multicellular marine taxa that alternatively employ both sexual and asexual reproduction^2^,^3^, either among specimen of a given species or between closely related species. The alternate reproductive traits present ecological opportunities (e.g., an increased propensity for successful genotypes) as well as ecological challenges (e.g., impacts of reduced genetic heterogeneity), and thus they may fundamentally determine the prospects for the propagule’s success. A hallmark difference between sexual/asexual reproductive traits is that while species that breed clonally (by fragmentation, budding, asexual propagules) can colonize and maintain similar habitats without requiring further local adaptation^4^, sexual reproduction may strongly influence the species’ adaptation to local environments and may also affect its distribution. In theory, asexual reproduction may further drive the population genetics of respective taxa away from classical expectations that are based on sexual-reproduction models; however, the empirical evidence available to date does not reveal sharp differences between sexually and asexually reproducing species^5^.

Ecological situations that guide asexual vs. sexual modes of reproduction in hermatypic corals have been the focus of scientific interest for many decades, as they directly shape the corals’ life-history strategies^6^,^7^. Corals display a diverse repertoire of asexual reproduction pathways, which includes fragmentation, polyp bail-out, budding and the production of asexual planula larvae (reviewed in^7^). Furthermore, even sexually derived floating embryos of broadcasting coral species may have the capacity for asexual expansion while split by turbulence into fragments, and then develop into numerous smaller asexual functional larvae (‘planktonic clones’^8^), a phenomenon that is induced in other marine invertebrates following specific biological stimuli^9^. The literature further shows that the sexual or asexual planktonic larval stage of reef corals facilitates species dispersal and habitat selection through a wide array of larval settlement traits and behaviors (reviewed by^6^,^7^), elucidating a critical factor in the ability of reef corals to invade new sites and/or to shape population recovery after major disturbances.

*Pocillopora damicornis*, a globally distributed branching coral species that inhabits a wide range of depths and habitats, is well known for its labile modes of reproduction, shifting from asexual pathways of fragmentation^10^ and parthenogenetic larvae^11^,^12^ to sexual reproduction via hermaphroditic broadcasting^12^,^13^,^14^, brooding^15^ or both, sometimes simultaneously^12^,^16^,^17^. As a brooder, *P. damicornis* may also sustain widely distributed populations by asexually producing planula larvae^11^,^12^,^18^,^19^. Although the production of asexual planulae leads to reduced genetic recombination compared to the production of sexual larvae, this can be counterbalanced by the simultaneous use of mixed sexual/asexual modes^12^,^16^,^18^,^20^. The use of mixed reproduction modes is recorded even within a single brood of *P. damicornis*^12^,^21^, resembling the results obtained for the hermaphroditic brooding congener *Seriatopora hystrix*^22^. In *P. damicornis*, genetic heterogeneity is also facilitated by the flow of infused genes by way of congener hybridization, by trans-oceanic dispersal via long competent larval periods^13^,^23^, or by the establishment of chimeric entities that develop from tissue fusions among densely settled recruites^24^,^25^. However, it is unclear whether the already explored sexual/asexual repertoire for *P. damicornis* reproduction reflects the entire spectrum of the reproductive modes characteristic of this species. In fact, the archetypal sets of the biological/environmental conditions that direct clonal versus sexual reproduction in *P. damicornis* are still ambiguous, despite the fact that it is one of the most extensively studied coral species.

After using microsatellite loci as markers of the genetic diversity of *P. damicornis* colonies that have originated from reefs in Thailand and the Philippines, we were surprised to find in both Indo-Pacific sites, a novel mode of asexual reproduction–chimeric larvae, originating from chimeric maternal colonies. Moreover, in a single clutch, released by a single specific colony, each of the chimeric larvae presented different combinations of maternal genotypic constituents, altogether creating a unique genetic variability by means of asexual reproduction, while also revealing the potential ecological advantages of this reproduction mode compared to commonly known sexual/asexual modes of reproduction.

## Results

**Figure 1:**
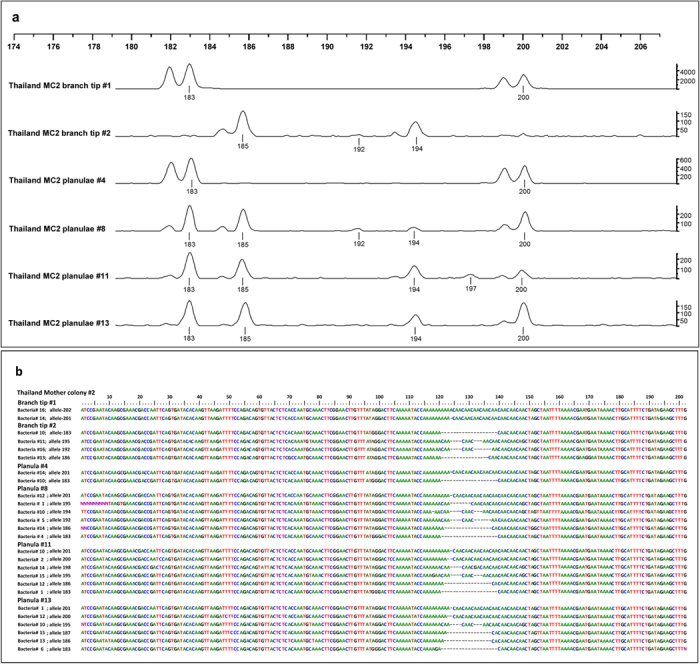
Sequencing microsatellite alleles from coral/planula DNA samples (Thai colony #2). (**a**) Microsatellite Pd3-002 profiles for samples from two branches from the mother colony (MC sample #1 and #2) and from four planulae (samples #4, #8, #11, #13). (**b**) Sequenced PCR products from the MC samples and from the four planulae. The numbers above the sequences indicate allele size (MC2, sample/planula #, bacterial clone #, allele size).

We collected 15–22 planulae from each of 12 mother colonies (six colonies from Thailand and six colonies from the Philippines) as well as two tissue samples that were taken from different branch tips in each mother colony. All samples were subjected to genetic analyses that assessed the genotypic diversity and the identity of the maternal colonies and their offspring using six polymorphic microsatellites (Table 1 and S1). Two major results emerged. The first was that the genotypic identities of released planulae mirrored the adult genotypes, alluding to the possibility of the asexual production of larvae in both Indo-Pacific reefs (Table S1). The second and most unexpected finding was that both the released larvae and their maternal colonies exhibited numerous cases of more than two alleles per locus. This phenomenon was repeated in four out of the six microsatellite loci (two loci in the Philippines: PV2 and PV6; and four loci in Thailand: PV2, PV6, Pd2-006 and Pd3-002). We considered these parent colonies and their asexually produced larvae to be chimeras, entities that simultaneously harbor cells that have originated from genetically distinct genotypes. This notion was further validated by the allele sequencing of six representative samples (Fig 1a,b and Figure S1, Table 1 and S1).

In the Philippines, chimerism was recorded in two loci, PV2 and PV6. In locus PV2, chimerism was found in all six larval clutches and in branch tips sampled from mother colonies (MC) Nos. 4 and 6. In locus PV6, chimerism was evident in the larval clutch released from MC3 (Table 1 and S1). In the two branch tips sampled from MC4, locus PV2 presented three alleles (133, 139, 161 bp), whereas the planula larvae released from the different branches of this mother colony exhibited three to four alleles each (i.e. the three alleles documented in the mother colony as well as allele 149 bp, which was characterized by lower peaks; these results were confirmed following repeated PCR amplifications). In Thailand, four out of the six microsatellites showed chimerism: it appeared in all six larval clutches from loci PV2 and PV6, and also in three of the larval clutches from loci Pd2-006 and Pd3-002 (Table 1). Results further revealed variable levels of chimeric contribution in each allele, as the outcomes for mother/offspring loci usually showed two major peaks and one or two different minor peaks (Table 1 and Table S1, Fig. 1a). Possible artifacts (e.g., PCR-induced chimeras) were ruled out by repeated PCR amplification and sequencing (Fig. 1a,b and Figure S1).

For six representative DNA samples (two mother colonies and four offspring), we cloned and sequenced *P. damicornis* microsatellite alleles from locus Pd3-002 (Fig. 1a,b and Figure S1, Table 1 and S1). Most of the bacterial clones (85/96) sent for sequencing produced good-quality sequences (Fig. S1). Interestingly, the two mother colony branch samples differed from each other (Fig. 1a,b): branch sample No. 1 showed two alleles, Nos. 183 and 201 bp. Branch sample No. 2, taken from the same colony, revealed two other major alleles, Nos. 186 and 195 bp, as well as the product of 192 bp. Of the four offspring arbitrarily selected released from this mother colony, one planula (No. 4) showed the same allelic pattern as branch sample No. 1, whereas the other three larvae (Nos. 8, 11 and 13) showed variable patterns from the mother colony’s allelic repertoire (alleles 183, 186, 195 and 201 bp). Planula No. 8 showed the 192-bp product in addition to alleles 183, 186, 195 and 201 bp; five alleles in total at microsatellite Pd3-002 (Fig. 1a,b).

It should be noted that not a single morphological indication (such as color differentiation, suture lines, or any unique/unusual pattern formation) was noticed during sampling of MCs to support the assumption that the chimeric colonies were a result of two colonies having fused together, rather than colonies having inherited the trait from their parents. In view of the above, while parent-colony chimerism is probably the outcome of tissue fusions between young colonies settling in close proximity (see Discussion), offspring chimerism is most likely the outcome of yet unknown budding processes from chimeric adult tissues, which contain a dissimilar genotypic distribution for each chimeric larva (Fig. 1a,b and Figure S1, Table 1 and S1).

## Discussion

For dispersion, coral reef organisms (including pocilloporid corals) rely on their motile planula stage, larvae that drift in the water column until they encounter a suitable substratum to settle on and metamorphose into primary polyps^6^,^7^. In the vast majority of cases this is a deterministic act, but in rare cases, stressed, newly metamorphosed pocilloporid primary polyps are capable of retracting soft tissues from their deposited skeleton and reverting to a motile ‘secondary larva’, which is capable of resettling and re-metamorphosing into another founder polyp^26^. The dispersal and ‘quality’ of the settling larvae, in pocilloporid species^27^ as in other reef-building corals, are therefore traits that are critical to the ability to invade new sites or to recover populations damaged by major disturbances.

Pursuant to the ongoing discussion about the significance of sexual/asexual modes of reproduction in reef-building organisms^26^, it is becoming evident that sexual and asexual lineages are important drivers in maintaining diverse coral populations and evolutionary processes^7^. Asexual reproduction produces genetically identical modules that may prolong the survival of adapted genotypes and induce wider dispersion, whereas sexual reproduction enables genetic recombination and the production of new coral genotypes that may enhance species fitness and survival. The decline in genetic heterogeneity that occurs following asexual reproduction can be compensated for by somatic mutations and by chimerism, two mechanisms that lead to genetically heterogeneous entities; the latter scenario may expose adjustable genotypic combinations of organismal traits to alterable and capricious natural selection operations^28^,^29^, thus enabling survivorship under harsh environmental conditions by giving rise to different expressed phenotypes that might each withstand a different selection pressure^29^.

Chimerism in sedentary marine organisms is associated with the trait of gregarious settlement^30^,^31^,^32^,^33^,^34^. A wealth of data on a range of scleractinian and alcyonarian coral species attests to the high frequency of gregarious larval set-ups, followed by allogeneic fusions^24^,^30^,^34^,^35^,^36^,^37^,^38^,^39^,^40^,^41^,^42^,^43^,^44^,^45^,^46^,^47^, the formation of hydrozoan chimeras through epithelial fusion between offspring polyps and adults^48^, the fusions between adult coral colonies and young settlers as a mechanism of wound healing^25^ and sometimes even between different yet closely related coral species or closely related genotypes^43^,^46^. As this phenomenon has been documented in a variety of sessile marine invertebrate phyla^33^,^49^, the occurrence of intraorganismal genetic variation in the field should not be considered an exceptional condition.

While organismal uniclonality is assumed to be functional and beneficial in preventing inner-organism conflicts^50^, established intraspecific chimerism in sessile, sedentary marine organisms may confer various ecological advantages, culminating in the formation of a ‘novel entity’ with a greater store of genetic variability and hence a potentially wider range of physiological qualities. Advantages include the establishment of an entity with an increased genetic repertoire, a reduced onset of reproduction, increased competitive capabilities during vulnerable juvenile stages, enhanced growth rates, reduced whole-colony mortality rates, synergistic complementation, and an assurance of mate location when needed^29^,^31^,^32^,^39^,^41^,^45^,^51^,^52^,^53^. Chimerism in sessile colonial marine organisms may also involve a trade-off mechanism that discerns between selection pressures at the individual and at the group levels^54^.

In light of the marked potential advantages of chimerism, the results of the present study challenge classical models and assumptions regarding dwindling genetic heterogeneity wherever asexual reproduction occurs. Our results, while excluding the possibility of parthenogenesis^11^, the ameiotic development of larvae from a single cell, reveal a novel avenue for asexual reproduction in corals: chimeric larval development by chimeric coral colonies, represented by a mosaicked expression of the participating genotypes. Even though the mechanism that produces these planulae is unknown, a budding process in chimeric adult tissues that develop into dissimilar genotypic contributions is the simplest explanation.

Asexual chimeric larvae may alter *P. damicornis* population genetics significantly (sensu^44^), serving as a tool to enhance intra-entity genetic heterogeneity without sex. Indeed, a recent study^55^ has revealed an excess of heterozygosity in *P. damicornis* populations when compared to the Hardy–Weinberg equilibrium expectations, which, following this study’s outcomes, may reflect the existence of chimeric larvae. Regardless of the various intrinsic fitness costs incurred with chimerism (such as cell competition/parasitism^33^), a chimera represents an alliance between partners that seems to offer advantages. Asexual chimeric larvae are more genetically diverse than sexually-derived larvae, presenting a higher per-capita rate of reproduction, as well as higher rates of genetic heterogeneity, and mitigating the loss of genetic diversity while also minimizing Allee effects in small colonizing populations. Most prominent are the benefits gained when chimeras change their somatic constituents, presenting the best fitted combination of their genetic components as a response to dissimilar environmental challenges^32^,^41^. In doing so, coral chimerism may also be used as a novel tool to combat impacts of global change. This is accomplished without the costs incurred by other routes, such as increased mutation rates^44^.

About 75 coral species are known to brood planula larvae and at least 10 additional species display mixed brooding/broadcasting modes of development^7^; all are potential candidates for the development of asexual chimeric larvae, a phenomenon further recorded in the sea anemone *Urticina felina*, which broods chimeric embryos and larvae^49^. Chimeras may also be developed through the fusions of embryos from broadcast spawning corals^47^.

The suggested performance exhibited by asexual chimeric larvae in *P. damicornis* may lead, in sites facing extreme ecological turnover and anthropogenic impacts like the Philippines and Thai reefs, to life-history traits that favor asexual modes of reproduction as the essential route for species dispersal. The aforementioned results as well the results on other coral chimeras^41^ further support the importance of the ‘group level’ (an assemblage of several genotypes in one entity) as the key tier for natural selection operations^25^,^46^. This tenet is further supported by the results of Mizrahi *et al.*^34^ regarding the mid-water metamorphosis of *Tubastraea coccinea* aggregated larvae (establishing chimeras; while not specified as such in the report), which showed a longer planktonic life and a greater dispersal potential, and by the results of Jiang *et al.*^47^ regarding fusions between embryos of the broadcast spawning coral *Platygyra daedalea*, which further developed into conjoined larvae.

Considering the functional ecological traits related to our findings, larval chimerism in *P. damicornis* may emerge as an operative tool for structuring and widening the species’ dispersal capabilities by exposing adjustable genotypic combinations of organismal traits to alterable natural selection operations, further shaping the interactions between the functions and the ecological traits, assisting *P. damicornis* with becoming a successful cosmopolitan species. The full extent and ecological significance of asexually produced planula larvae in corals that are released from chimeric maternal colonies as established chimera entities remains an open question that deserves additional detailed studies.

## Methods

### Material collection

Planulae were collected from 12 large (each 15–20 cm in diameter) gravid *P. damicornis* colonies, six from a reef near the Phuket Marine Station in Thailand (June 2006) and six from a reef near the Bolinao Marine Station, in the North Philippines (September 2006). The colonies were collected at each site from depths of 5–10 m and brought to the laboratory, where they were each placed in a separate aquarium supplied with running seawater and aeration and given a serial number (1–6 Maternal Colony [MC]). Coral colonies were kept in the aquaria for 1–2 nights, until 15–22 free-swimming planulae were released. Each planula was carefully collected with a syringe, placed in a separate 1.5 ml plastic vial and given its MC’s number followed by a sequential number. In addition, two small branch-tip pieces (0.5 cm) were removed with a small side cutter from different distanced branches of the same maternal colonies (labeled MC branch1, MC branch 2), before the colonies were returned to the collection sites. The side cutter and the other sampling tools were thoroughly washed and cleansed with alcohol and distilled water following each sampling.

### DNA extraction and PCR amplification

Genomic DNA was isolated from larvae and from maternal tissues following Graham^56^. Planulae and coral tissue samples were placed separately in plastic vials with a 200 μL lysis buffer (1 M Tris-borate pH 8.2, 0.5 M EDTA, 10% SDS, 5 M NaCl) and 40 μL of 5 M NaClO_4,_ shaken, supplemented with 240 μL phenol-chloroform-isoamyl alcohol (25:24:1, v/v), and then shipped to the IOLR laboratory in Israel. Samples were then centrifuged for 5 min at 14,000 rpm; the aqueous upper phase was collected and further mixed with 240 μL chloroform-isoamyl alcohol (24:1, v/v), centrifuged for 5 min at 14,000 rpm, and then the upper phase was collected and the DNA was precipitated with 500 μL of 100% cold ethanol overnight. Vials were centrifuged for 15 min at 4 °C, 14,000 rpm, washed with 500 μL of 70% ethanol and centrifuged for 5 min. Then the ethanol was removed and the precipitated DNA was dissolved in 40 μL DDW^56^.

*Microsatellite typing.* Six polymorphic microsatellite loci were used; loci PV2 and PV6^57^, loci Pd2-006, Pd3-008, Pd3-005, and locus Pd3-002^58^. All the PCR amplifications were carried out with fluorescently labeled primers in an Eppendorf thermocycler. Diluted DNA (1:50, 1 μL) from each sample was added to a reaction mixture containing 5 μM of each primer and DreamTaq™ DNA polymerase (Green PCR Master Mix 2×; Fermentas). The following PCR protocol was followed: 1 cycle at 94 °C for 1 min; 35 cycles at 94 °C for 1 min, 1 min at 50–55 °C and 1 min at 72 °C; 1 cycle at 72 °C for 10 min. The PCR products were screened on a 1.5% agarose gel to validate the amplification. The amplification products, which were at specific size ranges according to Magalon *et al.*^57^ and Starger *et al.*^58^, were sent for genotyping using an ABI PRISM 3100 Genetic Analyzer (Applied Biosystems).

### Data analysis

The amplification products were identified directly from the chromatographs using the Genotyper software version 3.7 (Applied Biosystems). The data were analyzed using the GenAlEx software version 6.2 59 and GENEPOP^60^. We identified an individual as a chimera if it was found to have more than 2 different alleles in a single microsatellite locus.

### Genotyping validation

All the samples which showed unusual allelic patterns (more than two alleles or no allele) were repeatedly tested 2 to 3 times in order to authenticate the PCR results. Samples indicating chimerism were re-amplified from the same DNA extract. A sample was considered to represent a chimera only if the second screen generated the same multilocus profile as the first. Studies on experimentally produced urochordate chimeras^61^ have revealed the critical importance of the relative genotypic distribution within a chimera. The cell numbers of a specific genotype reduced to below detectable levels (characteristic to the genotypic combination in each chimera, fluctuating from 1/10 to 1/100) yielded very weak or unscorable PCR products. As this study is based on a field-sampling protocol and not on chimeras created with the genotypic combination of known participants, the numbers of the microsatellite alleles detected in this study may be an underestimate of the whole allelic repertoire of the samples. Therefore, the numbers of scored alleles are conservative and the actual number of microsatellite alleles within the coral chimeras is unknown.

### Sequence cloning and analyses

As specified, an individual was considered a ‘chimera’ when the microsatellite profile consistently showed >2 alleles in a locus. To further verify chimerism, PCR products of the *P. damicornis* microsatellite Pd3-002 from two different branch samples of mother colony No. 2 (MC#2 Thailand) and from its four offspring (planula numbers 4,8,11 and 13) were cloned into a pDRIVE plasmid (Qiagene PCR cloning kit), which was then used to transform a XL1blue bacteria strain of *E. coli*. In total, 16 positive white bacteria colonies from each cloned plasmid were picked and sequenced using M13 forward and reveres universal primers. The sequences were analyzed and aligned with the GenBank *P. damicornis* Pd3-002 sequence (accession no. DQ684673; Fig. S1 first row) using the BioEdit software^62^.

## Additional Information

**How to cite this article**: Rinkevich, B. *et al.* Venturing in coral larval chimerism: a compact functional domain with fostered genotypic diversity. *Sci. Rep.* **6**, 19493; doi: 10.1038/srep19493 (2016).

## Supplementary Material

Supplementary Figure S1

## Figures and Tables

**Table 1:** Summary of all the microsatellite results from the branch tips (MC1 to MC6 for the Philippines and MC1 to MC6 for Thailand) and released planulae (column to the right of the specific MC results) of the 12 maternal colonies.

Philippines	MC 01	Planulae (20)	MC 02	Planulae (20)	MC 03	Planulae (20)	MC 04	Planulae (20)	MC 05	Planulae (20)	MC 06	Planulae (20)
PV2	Ht	Ch3 (18/20)	Ht	Ch3	Ht	Ch3	Ch3	Ch4	Ht	Ch3 (18/20)	Ch4	Ch4
		Ch4 (2/20)								Ch4 (2/20)		
PV6	Hm	Hm	Hm	Hm	Ht	Hm (2/20)	Ht	Ht	Ht	Hm (7/20)	Ht	Ht
						Ht (11/20)				Ht (13/20)		
						Ch3 (7/20)						
Pd3-002	Hm	Hm	Hm	Hm	Hm	Hm	Hm	Hm	Hm	Hm	Hm	Hm
Pd3-008	Ht	Ht	Ht	Ht	Ht	Ht	Hm	Hm	Ht	Ht	Ht	Ht
Pd2-006	Hm	Hm	Hm	Hm	Hm	Hm	Hm	Hm	Hm	Hm	Hm	Hm
Pd3-005	Ht	Ht	Ht	Ht	Ht	Ht	Ht	Ht	Ht	Ht	Ht	Ht
**Thailand**	**MC 01**	**Planulae (15)**	**MC 02**	**Planulae (20)**	**MC 03**	**Planulae (18)**	**MC 04**	**Planulae (20)**	**MC 05**	**Planulae(20)**	**MC 06**	**Planulae (22)**
PV2	Ch(3)	Hm (3/15)	Ht	Ht (6/20)	Hm	Hm (4/18)	Hm (1/2)	Ht (3/20)	Ht (1/2)	Ch3 (7/20)	*	Ch(3)
		Ht (8/15)		Ch3 (6/20)		Ht (2/18)	Ht (1/2)	Ch3 (17/20)	Ch3 (1/2)	Ch4 (13/20)		
		Ch3 (4/15)		Ch4 (8/20)		Ch3 (9/18)						
						Ch4 (3/18)						
PV6	Ht	Hm (5/15)	Ht	Hm (4/20)	Ht	Ht (14/18)	Ht	Hm (4/20)	Ht (1/2)	Ht (19/20)	*	Ht (4/22)
		Ht (7/15)		Ht (12/20)		Ch3 (4/18)		Ht (15/20)	Hm (1/2)	Ch3 (1/20)		Ch3 (18/22)
		Ch3 (3/15)		Ch3 (4/20)				Ch3 (1/20)				
Pd3-005	Hm	Hm	Hm	Hm	Ht	Ht	Hm	Hm	Hm	Hm	*	Ht
Pd3-008	Ht	Ht	Ht	Ht	Ht	Ht	Ht	Ht	Ht	Ht	*	Ht
Pd2-006	Ht	Hm (2/15)	Ht	Ht (19/20)	Hm	Hm	Ht	Hm (1/20)	Ht	Ht	*	Hm
		Ht (10/15)		Ch3 (1/20)				Ht (15/20)				
		Ch3 (2/15)						Ch3 (4/20)				
		Ch4 (1/15)										
Pd3-002	Ht	Ht	Ht	Ht (4/20)	Hm	Ht (2/18)	Hm (1/2)	Ht (6/20)	Ht	Ht	*	Ch3
				Ch3 (2/20)		Ch3 (15/18)	Ht (1/2)	Ch3 (8/20)				
				Ch4 (14/20)		Ch4 (1/18)		Ch4 (6/20)				

Hm = homozygote; Ht = heterozygote; Ch3, Ch4 = a chimera with 3 or 4 alleles/locus, respectively.

^*^Unsuccessful amplification (×3 PCRs).
