# Supplementary material for: Venturing in coral larval chimerism: a compact functional domain with fostered genotypic diversity
Source: Sci Rep. 2016 Jan 13;6:19493. doi: 10.1038/srep19493 (PMC4725755; doi:10.1038/srep19493)
Supplement: Supplementary Figure S1 [file srep19493-s1.pdf]

Venturing in coral larval chimerism: a compact functional domain with fostered genotypic diversity

Baruch Rinkevich[1]*, Lee Shaish[1,2], Jacob Douek,[1] Rachel Ben-Shlomo[3]

Supporting Information

Figure S1: Sequence analyses of 85 cloned PCR products from two tissue samples of the mother colony (MC #2; Thailand) and from the four offspring (MC2, branch tip sample/planula #, bacterial clone #, allele size). For summary of allele sizes, see Figure 1.

Table S1: Allele sizes (bp) in maternal colony (MC branch1, MC branch2) and offspring (p1–p22) samples. Each sample was recorded twice, independently. (–) no amplification results (×3 repetitions). Di nucleotides microsatellites (name, repeat tandem, no of repeats): PV2 $(CA)_{20}$, PV6 $(GT)_{7}$, Pd 2-006 $(CA)_{8}$; Tri- nucleotides microsatellites: Pd 3-008 $(CTG)_{7}$, Pd 3-005 $(TGA)_{9}$, Pd 3-002 $(AAC)_{10}$.

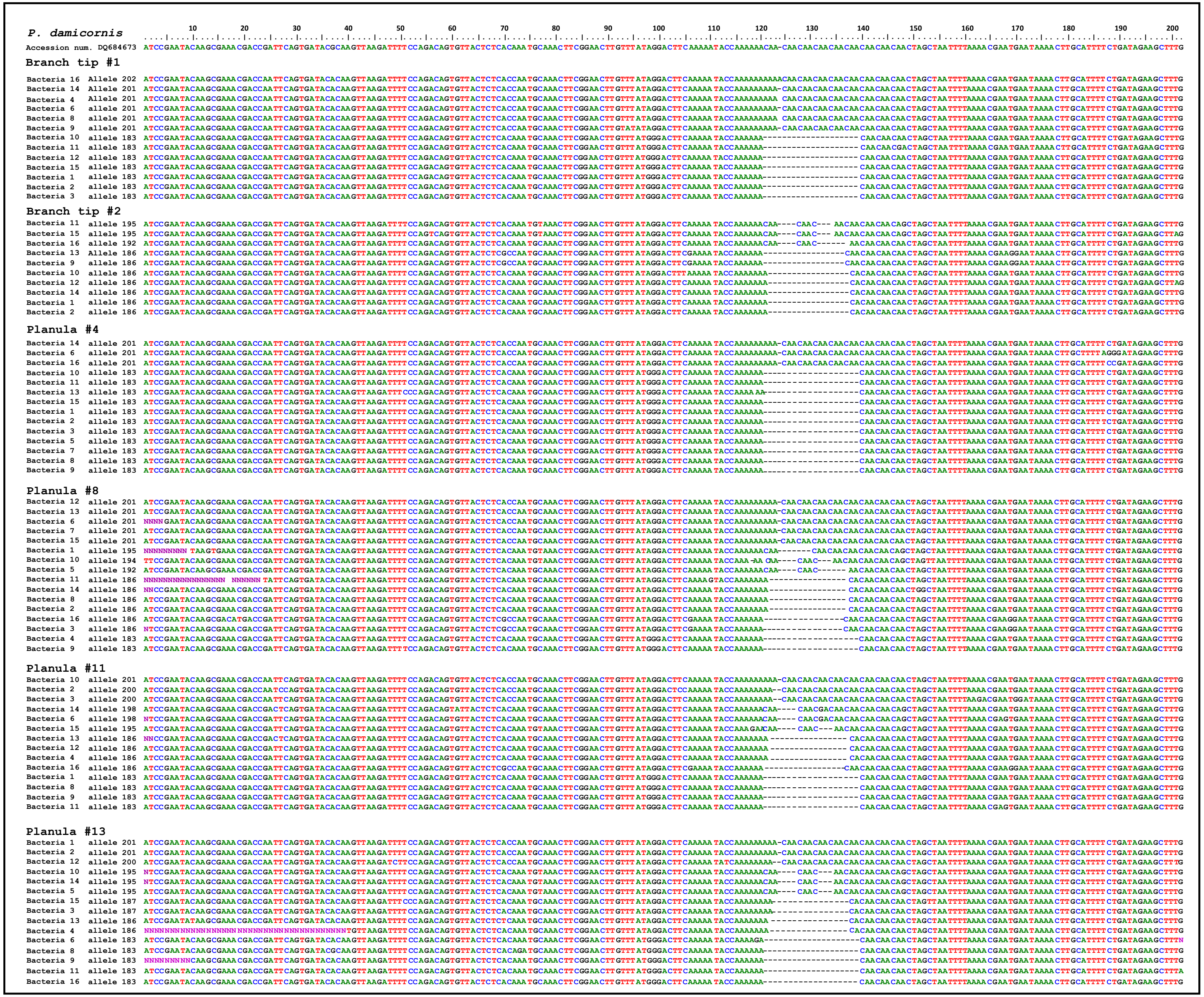
P. damicornis
Accession num. DQ684673
Branch tip #1
Branch tip #2
Planula #4
Planula #8
Planula #11
Planula #13

Table S1: Allele sizes (bp) in maternal colony.

Thailand Colony#	Sample	Locus Pd 3-008	Locus Pd 2-006	Locus Pd 3-005	Locus PV6	Locus PV2	Locus Pd 3-002
**1**	**MCbranch1**	162, 165	193,197	210	208,233	139,149,156	183,200
**1**	**MCbranch2**	162, 165	193,197	210	208,233	139,149,156	183,200
**1**	**p1**	162, 165	193	210	212	156	183,200
**1**	**p2**	162, 165	193,197	210	208,212	149,156	183,200
**1**	**p3**	162, 165	193,197	210	212	139,149,156	183,200
**1**	**p4**	162, 165	193,197	210	212	149,156	183,200
**1**	**p5**	162, 165	193,197	210	208,212,233	149,156	183,200
**1**	**p6**	162, 165	193,197	210	208,233	149,156	183,200
**1**	**p7**	162, 165	193,197	210	208,212,233	149,156	183,200
**1**	**p8**	162, 165	193,197	210	208,212	149,156	183,200
**1**	**p9**	162, 165	193,197	210	208,233	149,156	183,200
**1**	**p10**	162, 165	193,197,201	210	208	156	183,200
**1**	**p11**	162, 165	193,197,201	210	208	156	183,200
**1**	**p12**	162, 165	193,197	210	208,233	139,149,156	183,200
**1**	**p13**	162, 165	193,197	210	208,233	139,149	183,200
**1**	**p14**	162, 165	193,197,201,207	210	208,212,233	139,149,156	183,200
**1**	**p15**	162, 165	193	210	208,212	139,149,156	183,200
**2**	**MCbranch1**	162, 165	193,197	210	208,233	149,156	183,200
**2**	**MCbranch2**	162, 165	193,197	210	208,233	133,156	185,194
**2**	**p1**	162, 165	193,197	210	208,233	139,149,156	183,185,194,200
**2**	**p2**	162, 165	193,197	210	208,233	149,156	183,200
**2**	**p3**	162, 165	193,197,201	210	208,233	133,139,149,156	183,185,194,200
**2**	**p4**	162, 165	193,197	210	208,233	149,156	183,200
**2**	**p5**	162, 165	193,197	210	208,233	133,139,149,156	183,185,194,200
**2**	**p6**	162, 165	193,197	210	208,233	139,149,156	183,185,194,200
**2**	**p7**	162, 165	193,197	210	208,233	149,156	183,200
**2**	**p8**	162, 165	193,197	210	208,212,233	133,149,156	183,185,194,200
**2**	**p9**	162, 165	193,197	210	208	149,156	183,185,200
**2**	**p10**	162, 165	193,197	210	208	139,149,156	183,185,194,200
**2**	**p11**	162, 165	193,197	210	208,212	133,139,149,156	183,185,194,200
**2**	**p12**	162, 165	193,197	210	208,212,233	133,139,149,156	183,185,194,200
**2**	**p13**	162, 165	193,197	210	208	133,139,149,156	183,185,194,200
**2**	**p14**	162, 165	193,197	210	208,233	139,149,156	183,200
**2**	**p15**	162, 165	193,197	210	208,233	133,139,149,156	183,185,200
**2**	**p16**	162, 165	193,197	210	208,212	133,139,149,156	183,185,194,200
**2**	**p17**	162, 165	193,197	210	208,233	149,156	183,185,194,200
**2**	**p18**	162, 165	193,197	210	208	149,156	183,185,194,200
**2**	**p19**	162, 165	193,197	210	208,212,233	133,139,149,156	183,185,194,200

2	**p20**	162, 165	193,197	210	208,212,233	139,149,156	183,185,194,200
3	**MCbranch1**	-	-	-	-	-	-
3	**MCbranch2**	159, 162	197	213,219	212,215	133	183
3	**p1**	159, 162	197	213,219	206,208,210	133,139,149,156	185,194,200
3	**p2**	159, 162	197	213,219	208,210	133,156	185,194
3	**p3**	159, 162	197	213,219	208,210	156	185,194
3	**p4**	159, 162	197	213,219	208,212,215	133,149,156	183,185,194,200
3	**p5**	159, 162	197	213,219	212,215	133,139,156	183,196,200
3	**p6**	159, 162	197	213,219	208,212,215	133,139,156	183,185,200
3	**p7**	159, 162	197	213,219	212,215	133,139,149,156	183,196,200
3	**p8**	159, 162	197	213,219	208,212,215	133,139,149,156	183,196,200
3	**p9**	159, 162	197	213,219	212,215	133	183,196,200
3	**p10**	159, 162	197	213,219	212,215	133,156,161	183,196,200
3	**p11**	159, 162	197	213,219	212,215	133,149	183,196,200
3	**p12**	159, 162	197	213,219	212,215	133	183,196,200
3	**p13**	159, 162	197	213,219	212,215	133,139,149	183,196,200
3	**p14**	159, 162	197	213,219	212,215	133	183,196,200
3	**p15**	159, 162	197	213,219	212,215	133,139,149	183,196,200
3	**p16**	159, 162	197	213,219	212,215	133,139,149	183,196,200
3	**p17**	159, 162	197	213,219	212,215	133,139,149	183,196,200
3	**p18**	159, 162	197	213,219	212,215	133,139,149	183,196,200
4	**MCbranch1**	162, 165	193,197	210	208,233	156	183
4	**MCbranch2**	162, 165	193,197	210	208,233	149,156	183,200
4	**p1**	162, 165	193,197	210	208,212	139,149,156	183,185,194,200
4	**p2**	162, 165	193	210	208,212	133,149,156	183,185,200
4	**p3**	162, 165	193,197,200	210	208	133,149,156	183,185,194
4	**p4**	162, 165	193,197	210	208,212	133,149,156	183,185,194,200
4	**p5**	162, 165	193,197	210	208,212	133,149,156	185,194
4	**p6**	162, 165	193,197	210	208,233	149,156	183,185,194,200
4	**p7**	162, 165	193,197,200	210	208	133,149,156	185,194,200
4	**p8**	162, 165	193,197	210	208	133,149,156	185,194,200
4	**p9**	162, 165	193,197	210	208,233	139,149,156	183,200
4	**p10**	162, 165	193,197	210	208,212,233	139,149,156	183,185,194,200
4	**p11**	162, 165	193,197	210	208,233	139,149,156	183,200
4	**p12**	162, 165	193,197	210	208,233	139,149,156	183,200
4	**p13**	162, 165	193,197	210	208,233	149,156	183,185,194,200
4	**p14**	162, 165	193,197	210	208,233	149,156	183,200
4	**p15**	162, 165	193,197	210	208,233	133,139,149	183,185,194,200
4	**p16**	162, 165	193,197	210	208,212	139,149,156	183,200
4	**p17**	162, 165	193,197	210	208,212	139,149,156	183,185,200
4	**p18**	162, 165	193,197	210	208	139,149,156	183,185,200
4	**p19**	162, 165	193,197,200	210	208,233	139,149,156	183,185,200

**4**	**p20**	162, 165	193,197,200	210	208,233	139,149,156	183,194,200
**5**	**MCbranch1**	162, 165	193,197	210	208,233	139,149,156	183,200
**5**	**MCbranch2**	162, 165	193,197	210	208	149,156	183,200
**5**	**p1**	162, 165	193,197	210	208,233	139,149,156	183,200
**5**	**p2**	162, 165	193,197	210	208,233	133,139,149,156	183,200
**5**	**p3**	162, 165	193,197	210	208,233	133,149,156	183,200
**5**	**p4**	162, 165	193,197	210	208,233	133,139,149,156	183,200
**5**	**p5**	162, 165	193,197	210	208,233	133,139,149,156	183,200
**5**	**p6**	162, 165	193,197	210	208,233	133,149,156	183,200
**5**	**p7**	162, 165	193,197	210	208,233	133,139,149,156	183,200
**5**	**p8**	162, 165	193,197	210	208,233	133,149,156	183,200
**5**	**p9**	162, 165	193,197	210	208,233	133,139,149,156	183,200
**5**	**p10**	162, 165	193,197	210	208,233	133,139,149,156	183,200
**5**	**p11**	162, 165	193,197	210	208,233	133,139,149,156	183,200
**5**	**p12**	162, 165	193,197	210	208,233	133,149,156	183,200
**5**	**p13**	162, 165	193,197	210	208,233	133,139,149,156	183,200
**5**	**p14**	162, 165	193,197	210	208,233	133,139,149,156	183,200
**5**	**p15**	162, 165	193,197	210	208,212,233	133,149,156	183,200
**5**	**p16**	162, 165	193,197	210	208,233	133,139,149,156	183,200
**5**	**p17**	162, 165	193,197	210	208,233	133,149,156	183,200
**5**	**p18**	162, 165	193,197	210	208,233	133,139,149,156	183,200
**5**	**p19**	162, 165	193,197	210	208,233	133,139,149,156	183,200
**5**	**p20**	162, 165	193,197	210	208,233	133,139,149,156	183,200
**6**	**MCbranch1**	-	-	-	-	-	-
**6**	**MCbranch2**	-	-	-	-	-	-
**6**	**p1**	159, 162	197	213,219	208,212,215	133,139,149	183,196,200
**6**	**p2**	159, 162	197	213,219	208,212,215	133,139,149	183,196,200
**6**	**p3**	159, 162	197	213,219	208,212,215	133,139,149	183,196,200
**6**	**p4**	159, 162	197	213,219	208,212	133,139,149	183,196,200
**6**	**p5**	159, 162	197	213,219	208,212,215	133,139,149	183,196,200
**6**	**p6**	159, 162	197	213,219	208,212,215	133,139,149	183,196,200
**6**	**p7**	159, 162	197	213,219	208,212,215	133,139,149	183,196,200
**6**	**p8**	159, 162	197	213,219	208,212,215	133,139,149	183,196,200
**6**	**p9**	159, 162	197	213,219	208,212,215	133,139,149	183,196,200
**6**	**p10**	159, 162	197	213,219	208,212,215	133,139,149	183,196,200
**6**	**p11**	159, 162	197	213,219	208,212,215	133,139,149	183,196,200
**6**	**p12**	159, 162	197	213,219	208,212	133,139,149	183,196,200
**6**	**p13**	159, 162	197	213,219	208,212,215	133,139,149	183,196,200
**6**	**p14**	159, 162	197	213,219	208,212,215	133,139,149	183,196,200
**6**	**p15**	159, 162	197	213,219	208,212,215	133,139,149	183,196,200
**6**	**p16**	159, 162	197	213,219	208,212	133,139,149	183,196,200
**6**	**p17**	159, 162	197	213,219	208,212	133,139,149	183,196,200

**6**	**p18**	159, 162	197	213,219	208,212,215	133,139,149	183,196,200
**6**	**p19**	159, 162	197	213,219	208,212,215	133,139,149	183,196,200
**6**	**p20**	159, 162	197	213,219	208,212,215	133,139,149	183,196,200
**6**	**p21**	159, 162	197	213,219	208,212,215	133,139,149	183,196,200
**6**	**p22**	159, 162	197	213,219	208,212,215	133,139,149	183,196,200
**Philippines**		**Locus**	**Locus**	**Locus**	**Locus**	**Locus**	**Locus**
**Colony#**	**Sample**	**Pd 3-008**	**Pd 2-006**	**Pd 3-005**	**V6**	**V2**	**Pd 3002**
**1**	**MCbranch1**	162, 165	193	219,222	208	149,161	184
**1**	**MCbranch2**	162, 165	193	219,222	208	149,161	184
**1**	**p1**	162, 165	193	219,222	208	139,149,161	184
**1**	**p2**	162, 165	193	219,222	208	139,149,161	184
**1**	**p3**	162, 165	193	219,222	208	139,149,161	184
**1**	**p4**	162, 165	193	219,222	208	139,149,161	184
**1**	**p5**	162, 165	193	219,222	208	139,149,161	184
**1**	**p6**	162, 165	-	219,222	208	139,149,161	184
**1**	**p7**	162, 165	193	219,222	208	139,149,161	184
**1**	**p8**	162, 165	193	219,222	208	139,149,161	184
**1**	**p9**	162, 165	193	219,222	208	139,149,161	184
**1**	**p10**	162, 165	193	219,222	208	139,149,161	184
**1**	**p11**	162, 165	193	219,222	208	139,149,161	184
**1**	**p12**	162, 165	193	219,222	208	139,149,161	184
**1**	**p13**	162, 165	193	219,222	208	139,149,161	184
**1**	**p14**	162, 165	193	219,222	208	139,149,161	184
**1**	**p15**	162, 165	193	219,222	208	139,149,161	184
**1**	**p16**	162, 165	193	219,222	208	135,139,149,161	184
**1**	**p17**	162, 165	193	219,222	208	135,139,149,161	184
**1**	**p18**	162, 165	193	219,222	208	139,149,161	184
**1**	**p19**	162, 165	193	219,222	208	139,149,161	184
**1**	**p20**	162, 165	193	219,222	208	139,149,161	184
**2**	**MCbranch1**	-	193	-	208	-	184
**2**	**MCbranch2**	162, 165	193	219,222	208	149,161	184
**2**	**p1**	162, 165	193	219,222	208	139,149,161	184
**2**	**p2**	162, 165	193	219,222	208	139,149,161	184
**2**	**p3**	162, 165	193	219,222	208	139,149,161	184
**2**	**p4**	162, 165	193	219,222	208	139,149,161	184
**2**	**p5**	162, 165	193	219,222	208	139,149,161	184
**2**	**p6**	162, 165	193	219,222	208	139,149,161	184
**2**	**p7**	162, 165	193	219,222	208	139,149,161	184
**2**	**p8**	162, 165	193	219,222	208	139,149,161	184
**2**	**p9**	162, 165	193	219,222	208	139,149,161	184
**2**	**p10**	162, 165	193	219,222	208	139,149,161	184
**2**	**p11**	162, 165	193	219,222	208	139,149,161	184

**2**	**p12**	162, 165	193	219,222	208	139,149,161	184
**2**	**p13**	162, 165	193	219,222	208	139,149,161	184
**2**	**p14**	162, 165	193	219,222	208	139,149,161	184
**2**	**p15**	162, 165	193	219,222	208	139,149,161	184
**2**	**p16**	162, 165	193	219,222	208	139,149,161	184
**2**	**p17**	162, 165	193	219,222	208	139,149,161	184
**2**	**p18**	162, 165	193	219,222	208	139,149,161	184
**2**	**p19**	162, 165	193	219,222	-	139,149,161	184
**2**	**p20**	162, 165	193	219,222	208	139,149,161	184
**3**	**MCbranch1**	162, 165	193	219,222	208,212	149,161	184
**3**	**MCbranch2**	162, 165	193	219,222	208,212	149,161	184
**3**	**p1**	162, 165	193	219,222	208,231	139,149,161	184
**3**	**p2**	162, 165	193	219,222	208,212,231	139,149,161	184
**3**	**p3**	162, 165	193	219,222	208,212	139,149,161	184
**3**	**p4**	162, 165	193	219,222	208,212,231	139,149,161	184
**3**	**p5**	162, 165	193	219,222	208	139,149,161	184
**3**	**p6**	162, 165	193	219,222	208,212	139,149,161	184
**3**	**p7**	162, 165	193	219,222	208,212	139,149,161	184
**3**	**p8**	-	-	-	208,231	139,149,161	184
**3**	**p9**	162, 165	193	219,222	208,212,231	139,149,161	184
**3**	**p10**	162, 165	193	219,222	208,212	139,149,161	184
**3**	**p11**	162, 165	193	219,222	208,231	139,149,161	184
**3**	**p12**	162, 165	193	219,222	208,212,231	139,149,161	184
**3**	**p13**	162, 165	193	219,222	208,212,231	139,149,161	184
**3**	**p14**	162, 165	193	219,222	208	139,149,161	184
**3**	**p15**	162, 165	193	219,222	208,212	139,149,161	184
**3**	**p16**	-	-	-	208,212,231	139,149,161	184
**3**	**p17**	162, 165	193	219,222	208,231	139,149,161	184
**3**	**p18**	162, 165	193	219,222	208,231	139,149,161	184
**3**	**p19**	162, 165	193	219,222	208,231	139,149,161	184
**3**	**p20**	162, 165	193	219,222	208,212,231	139,149,161	184
**4**	**MCbranch1**	162	197	216,219	208,212	133,139,161	181
**4**	**MCbranch2**	162	197	216,219	208,212	133,139,161	181
**4**	**p1**	162	197	216,219	208,212	133,139,149,161	181
**4**	**p2**	162	197	216,219	208,212	133,139,149,161	181
**4**	**p3**	162	197	216,219	208,212	133,139,149,161	181
**4**	**p4**	162	197	216,219	208,212	133,139,149,161	181
**4**	**p5**	162	197	216,219	208,212	133,139,149,161	181
**4**	**p6**	162	197	216,219	208,212	133,139,149,161	181
**4**	**p7**	162	197	216,219	208,212	133,139,149,161	181
**4**	**p8**	162	197	216,219	208,212	133,139,149,161	181
**4**	**p9**	162	197	216,219	208,212	133,139,149,161	181

**4**	**p10**	162	197	216,219	208,212	133,139,149,161	181
**4**	**p11**	162	197	216,219	208,212	133,139,149,161	181
**4**	**p12**	162	197	216,219	208,212	133,139,149,161	181
**4**	**p13**	162	197	216,219	208,212	133,139,149,161	181
**4**	**p14**	162	197	216,219	208,212	133,139,149,161	181
**4**	**p15**	162	197	216,219	208,212	133,139,149,161	181
**4**	**p16**	162	197	216,219	208,212	133,139,149,161	181
**4**	**p17**	162	197	216,219	208,212	133,139,149,161	181
**4**	**p18**	162	197	216,219	208,212	133,139,149,161	181
**4**	**p19**	162	197	216,219	208,212	133,139,149,161	181
**4**	**p20**	162	197	216,219	208,212	133,139,149,161	181
**5**	**MCbranch1**	162, 165	197	222,237	208,212	133,149	181
**5**	**MCbranch2**	162, 165	197	222,237	208,212	133,149	181
**5**	**p1**	162, 165	197	222,237	208,212	133,139,149	181
**5**	**p2**	162, 165	197	222,237	208	133,139,149	181
**5**	**p3**	162, 165	197	222,237	208,212	133,139,149	181
**5**	**p4**	162, 165	197	222,237	208	133,139,149	181
**5**	**p5**	162, 165	197	222,237	208,212	133,139,149	181
**5**	**p6**	162, 165	197	222,237	208	133,139,149	181
**5**	**p7**	162, 165	197	222,237	208	133,139,149	181
**5**	**p8**	162, 165	197	222,237	208,212	133,139,149	181
**5**	**p9**	162, 165	197	222,237	208,212	133,139,149	181
**5**	**p10**	162, 165	197	222,237	208,212	133,139,149	181
**5**	**p11**	162, 165	197	222,237	208,212	133,139,149,156	181
**5**	**p12**	162, 165	197	222,237	208	133,139,149	181
**5**	**p13**	162, 165	197	222,237	208,212	133,139,149	181
**5**	**p14**	162, 165	197	222,237	208,212	133,139,149	181
**5**	**p15**	162, 165	197	222,237	208,212	133,139,149	181
**5**	**p16**	162, 165	197	222,237	208,212	133,139,149	181
**5**	**p17**	162, 165	197	222,237	208,212	133,139,149,156	181
**5**	**p18**	162, 165	197	222,237	208	133,139,149	181
**5**	**p19**	162, 165	197	222,237	208	133,139,149	181
**5**	**p20**	162, 165	197	222,237	208,212	133,139,149	181
**6**	**MCbranch1**	159, 162	197	212,243	208,212	133,139,149,161	184
**6**	**MCbranch2**	159, 162	197	212,243	208,212	133,139,149,161	184
**6**	**p1**	159, 162	197	212,243	208,212	133,139,149,161	184
**6**	**p2**	159, 162	197	212,243	208,212	133,139,149,161	184
**6**	**p3**	159, 162	197	212,243	208,212	133,139,149,161	184
**6**	**p4**	159, 162	197	212,243	208,212	133,139,149,161	184
**6**	**p5**	159, 162	197	212,243	208,212	133,139,149,161	184
**6**	**p6**	159, 162	197	212,243	208,212	133,139,149,161	184
**6**	**p7**	159, 162	197	212,243	208,212	133,139,149,161	184

**6**	**p8**	159, 162	197	212,243	208,212	133,139,149,161	184
**6**	**p9**	159, 162	197	212,243	208,212	133,139,149,161	184
**6**	**p10**	159, 162	197	212,243	208,212	133,139,149,161	184
**6**	**p11**	159, 162	197	212,243	208,212	133,139,149,161	184
**6**	**p12**	159, 162	197	212,243	208,212	133,139,149,161	184
**6**	**p13**	159, 162	197	212,243	208,212	133,139,149,161	184
**6**	**p14**	159, 162	197	212,243	208,212	133,139,149,161	184
**6**	**p15**	159, 162	197	212,243	208,212	133,139,149,161	184
**6**	**p16**	159, 162	197	212,243	208,212	133,139,149,161	184
**6**	**p17**	159, 162	197	212,243	208,212	133,139,149,161	184
**6**	**p18**	159, 162	197	212,243	208,212	133,139,149,161	184
**6**	**p19**	159, 162	197	212,243	208,212	133,139,149,161	184
**6**	**p20**	159, 162	197	212,243	208,212	133,139,149,161	184

Allele sizes (bp) in maternal colony (MC branch1, MC branch2) and offspring (p1–p22) samples. Each sample was recorded twice, independently. (–) no amplification results (×3 repetitions).
